# Variation in the Sweet Taste Receptor Gene and Dietary Intake in a Swedish Middle-Aged Population

**DOI:** 10.3389/fendo.2017.00348

**Published:** 2017-12-13

**Authors:** Caroline Habberstad, Isabel Drake, Emily Sonestedt

**Affiliations:** ^1^Nutritional Epidemiology, Department of Clinical Sciences Malmö, Lund University, Malmö, Sweden; ^2^Diabetes and Cardiovascular Disease – Genetic Epidemiology, Department of Clinical Sciences Malmö, Lund University, Malmö, Sweden

**Keywords:** taste receptor gene, sweet taste receptor gene, polymorphism, diet, cohort, epidemiology

## Abstract

**Background:**

The preference for sweet taste is partially genetically determined. The major allele of the single nucleotide polymorphism rs12033832 in the sweet taste receptor (*TAS1R2*) has previously been associated with lower sugar sensitivity and higher sugar intake among overweight individuals. The aim of the present study was to examine the association between dietary intake and the *TAS1R2* genotype in lean and overweight individuals in the population-based Malmö Diet and Cancer (MDC) cohort using dietary intake data with a high validity.

**Methods:**

In total, 3,602 participants (46–68 years old) from the MDC cohort who underwent baseline examinations between 1991 and 1994, who were non-smokers without diabetes, and for whom information regarding *TAS1R2* rs7534618 (a proxy for rs12033832) was available were included in this study. After excluding individuals with potentially misreported and unstable food habits, 2,204 individuals were retained. A modified dietary history method, including a 7-day food diary of prepared meals, which was specifically designed for the MDC study was used.

**Results:**

Only modest associations were observed between dietary intake and the *TAS1R2* genotype. We observed slightly stronger associations after excluding individuals with potentially misreported and unstable food habits. Among the participants with a BMI ≥25, the major (T) allele carriers consumed more carbohydrates [TT = 45.2 percentage of energy intake (E%); TG = 45.2E%; GG = 43.7E%; *p* = 0.01] and less fat (*p* = 0.03), but these participants did not consume more sucrose than the G-allele carriers. No association was observed between the genotype and dietary intake among the participants with a BMI <25.

**Conclusion:**

Although the higher carbohydrate intake among the major allele carriers was consistent with that reported in a previous study, the magnitudes of the associations were substantially smaller. Because we observed no association with sucrose, this allele is unlikely to be useful as a marker of sugar intake in the MDC population.

## Introduction

The taste sensory system is complex and involves multiple steps from the taste cell receptors on the tongue to finally experiencing the conscious perception of taste. The sweet taste is mainly mediated by a heterodimer of the taste receptors type 1 member 2 (TAS1R2) and member 3 (TAS1R3) (1). Because TAS1R3 can also form a heterodimer with TAS1R1 and convey the taste of umami (1), *TAS1R2* is most relevant to conveying the sweet taste. Individual differences exist in the sensitivity, perception, and preference for tastes. Sensitivity to a particular taste is due to the threshold of taste cells activated. Although the sensitivity is largely assumed to be genetically determined (2–8), a few studies have examined the relationship between variations in the *TAS1R2* gene and sweetness perception and sugar consumption. In a study involving 95 individuals, a link to suprathreshold sweet taste sensitivity (assessed by participants’ ratings of the sucrose intensity of five solutions using general Labeled Magnitude Scales) was found in two of eight examined genetic variants (tag SNPs) in *TAS1R2* (9). These two genetic variants were moderately linked (*r*^2^ = 0.67), and only one variant (rs12033832) was also associated with dietary intake (based on a food frequency questionnaire) in a larger study involving 707 individuals. However, the relationship between rs12033832 (G > A) and sugar intake and sensitivity to sweet taste was dependent on BMI. Among the participants with a BMI of 25 or above, G allele carriers had a lower sensitivity, a higher threshold (as determined by a staircase procedure in which the participants were tasked with identifying the sucrose solution among three solutions), and consumed more carbohydrates (277 vs. 214 g/day), including sucrose (50 vs. 36 g/day), than the non-carriers. Among the participants with a BMI below 25, the G allele carriers had a lower threshold and consumed fewer carbohydrates. Fushan et al. (4) found no significant relationship between rs12033832 and sensitivity to sugar; however, BMI was not considered in this study.

The purpose of this study was to examine the relationship between the SNP rs12033832 in *TAS1R2* and dietary intake in a larger, older, population-based cohort, i.e., the Malmo Diet and Cancer Study cohort. Because decreased sensitivity to sugar might lead to increased intake of foods with a high sugar content, we aimed to investigate whether the dietary composition differed among those with genotypes associated with a decreased sensitivity to sweet taste. If the correlation between increased intake of sugar and the *TASIR2* variant is replicated and validated, this variant could be used as an objective marker for increased sugar intake in future studies.

## Subjects and Methods

### Study Participants and Data Collection

The Malmo Diet and Cancer (MDC) study cohort includes men born between 1923 and 1945 and women born between 1923 and 1950 living in Malmo (10). The recruitment was conducted between March 1991 and October 1996 *via* personal letters or public advertising. The exclusion criteria included participants with a limited knowledge of Swedish and mentally impaired individuals. The participants signed a written consent form, and ethical permission is available for the Malmo Diet and Cancer study (LU 90-51). All individuals visited the study center twice. During the first visit, the participants received information about the study and the dietary assessment method. A comprehensive standardized questionnaire was handed out, and the participants were asked to answer questions regarding their physical activity habits, social networks, occupation, use of tobacco, medical history, diseases of close relatives, demographic variables, etc. During the first visit, trained personnel collected blood samples, and anthropometrics were obtained. During the second visit, which occurred approximately 10 days after the first visit, an individual interview was performed to collect the final components of the diet history and verify that the lifestyle questionnaire was completed correctly. In total, 28,098 individuals (60% women) completed the lifestyle questionnaire, underwent anthropometric measurements, and participated in the diet assessment. Between November 1991 and February 1994, 6,103 participants were randomly selected to participate in a cardiovascular subcohort. Most of these participants underwent several measurements, including measurements of glucose, insulin, triglycerides, and high-density lipoprotein cholesterol (HDL-C) levels from fasting blood samples. The participants were also genotyped (see below). In total, information regarding the *TAS1R2* genotype and dietary intake was available for 5,137 individuals. Because smoking may alter the taste experience (11), we excluded smokers (*n* = 1,413). After further excluding individuals with self-reported diabetes medication and diagnosis (*n* = 119) and those without BMI data (*n* = 3), 3,602 individuals remained in the study. After excluding the diet changers (25.5%) and inadequate energy reporters (19.1%), 2,204 participants were retained.

### Genotyping

An Illumina OmniExpress (Illumina, San Diego, CA, USA) was used to genotype the entire genome. We used a proxy (rs7534618) that is highly linked to rs12033832 (*r*^2^ = 0.869; *D*’ = 1.000). This SNP was in Hardy–Weinberg equilibrium in the studied population (*p* = 0.22). The minor allele frequency of the G allele was 0.37.

### Dietary Data

To collect information regarding the participants’ dietary habits, we used a diet history method that combined a 7-day food diary, a 168-item food questionnaire, and a 1-h interview. This method of collecting dietary data was designed specifically for the MDC cohort (12, 13). In the food diary, the participants recorded their cooked meals (usually lunch and dinner meals) and cold beverages. In the food questionnaire, the participants estimated the average frequency and portion size of the foods consumed during the previous year. In the interview, the participants answered questions about their food choices, how they performed their cooking, and the average portion sizes of the meals in the food diary. The interviewer also verified that the information in the questionnaire and food diary did not overlap. The MDC food and nutrient database, which was specifically developed for this study, was based on the PC diet version 2/93 by the National Food Agency. The information in the database was used to transform the daily food intake (from the questionnaire and diary) to the daily intake of nutrients. The following dietary variables were examined in this study: (1) total energy intake (kcal/day); (2) percentage of non-alcohol energy intake from fat, carbohydrates, monosaccharides (fructose and glucose), disaccharides (lactose, sucrose and maltose), sucrose, and protein; and (3) fiber density (g fiber/1,000 kcal total energy). We also examined 29 food intake variables (g/day) that cover the whole diet and specifically separate foods based on the sugar content. In the validation study, which comprised 241 Malmö residents (126 men and 115 women) aged between 50 and 69 years, the validity of the diet method was evaluated by comparing this method to the weighted food intake information on 18 separate days during a 1-year period (12, 14). The validity was generally high with the following energy-adjusted Pearson correlation coefficients: fat (women: 0.69/men: 0.64), protein (0.53/0.54), carbohydrates (0.70/0.66), sucrose (0.74/0.60), and fiber (0.69/0.74). Seasonal variations in dietary habits were considered, and a variable was created for winter, spring, summer, and autumn.

### Statistical Analyses

SPSS version 23 (IBM Corporation, Armonk, NY, USA) was used for all statistical analyses. We performed a Chi-squared analysis of the categorical variables and a generalized linear model to analyze the log-transformed continuous dietary variables to examine the association between the variables and the BMI groups (<25 and ≥25), and between the variables and genotype stratified by the BMI groups. The analysis of the dietary factors was adjusted for age, sex, energy intake, and season. The heterogeneity of the effect was evaluated by including a multiplicative factor (genotype × BMI) in the model. In the sensitivity analysis, we excluded individuals with potentially misreported energy intake (15) and individuals who had substantially changed their diet in the past and, therefore, may have unstable food habits (16, 17). The misreporters were identified by comparing the estimated energy intake with the estimated energy expenditure. The individuals were categorized as having changed their diet in the past if they answered yes to the following questionnaire item, “Have you substantially changed your eating habits because of illness or for some other reason?”

## Results

### Study Population

The study sample included 3,602 non-smoking individuals, and 40% of the participants were males; the average age was 57 years (ranging from 48 to 68 years). In this population, 41% of the participants were homozygous for the T allele, 46% of the participants were heterozygous (GT), and 14% of the participants were homozygous for the G allele. In total, 55% of the participants had a BMI of 25 or above, 26% of the participants reported a substantial diet change in the past, and 19% of the participants were classified as being inadequate energy reporters. Table 1 shows the dietary intake across the BMI groups in the study sample excluding the diet changers and inadequate energy reporters (*n* = 2,204).

**Table 1:** Participant characteristics according to the BMI categories after excluding the potential misreporters and individuals with unstable food habits.

	BMI <25 (*n* = 1,049)	BMI ≥25 (*n* = 1,155)	*p*-Value
Energy, kcal/day	2,389 (16)	2,389 (16)	0.97
Carbohydrates, E%	45.6 (0.2)	45.1 (0.2)	0.04
Monosaccharides, E%	7.8 (0.1)	7.5 (0.1)	0.02
Disaccharides, E%	12.7 (0.1)	12.9 (0.1)	0.18
Sucrose, E%	8.6 (0.1)	8.6 (0.1)	0.89
Fiber, g/1,000 kcal	9.3 (0.1)	9.0 (0.1)	0.01
Fat, E%	39.2 (0.2)	39.3 (0.2)	0.63
Protein, E%	15.3 (0.1)	15.7 (0.1)	<0.001

*Numbers represent the means (SE). A general linear model adjusting for age, sex, total energy intake, and season was used to evaluate the differences among the groups*.

### Association between the *TAS1R2* Genotype and Dietary Intake

A statistically significant association was observed between the *TAS1R2* genotype and carbohydrate intake among individuals with a BMI of 25 or above (Table S1 in Supplementary Material). The proportion of participants who reported that they changed their diets in the past differed among the genotypes in the participants with a BMI ≥ 25 (Table S1 in Supplementary Material). When diet changers and inadequate energy reporters were excluded, the individuals with the GT or TT genotype and a BMI ≥ 25 reported a higher consumption of carbohydrates (*p* = 0.01) and a lower consumption of fat (*p* = 0.03) than those with the GG genotype (Table 2). A high correlation was observed between carbohydrate and fat intake (Pearson correlation coefficient = −0.93). The interaction between genotype and BMI groups on the dietary intakes did not reach the level of statistically significance (*p*-interaction = 0.15 and 0.20 for carbohydrates and fat, respectively). The individuals with the TT or GT genotype consumed an average of 45.2% of their energy intake from carbohydrates, whereas those with the GG genotype consumed 43.7% of their energy intake from carbohydrates. This consumption corresponded to a reported intake of 260 and 251 g/day of carbohydrates among the individuals with the TT or GT genotype and individuals with the GG genotype, respectively. No differences were observed in the sucrose intake across the genotypes (*p* = 0.51); however, a marginally significant lower disaccharide intake (*p* = 0.06) and lower intake of dairy products containing lactose (non-fermented milk and cream) were associated with the G-allele (Table 3). In addition, we observed a lower intake of potatoes and margarine and a higher intake of fiber-rich bread and cheese among the G-allele carriers with a BMI of 25 or above. Moreover, no significant association was observed between the macronutrient intake and the genotype, but a higher intake of non-fermented milk was observed among the G-allele carriers, in the group with a BMI below 25.

**Table 2:** Associations between the *TAS1R2* genetic variant (rs7534618) and dietary intake after excluding the potential misreporters and individuals with unstable food habits.

	BMI <25				BMI ≥25			
	TT (*n* = 445)	GT (*n* = 459)	GG (*n* = 145)	*p*-Trend	TT (*n* = 470)	GT (*n* = 525)	GG (*n* = 160)	*p*-Trend
Energy, kcal/day	2,338 (23)	2,366 (23)	2,333 (41)	0.76	2,434 (24)	2,413 (23)	2,433 (41)	0.88
Carbohydrates, E%	45.7 (0.3)	45.5 (0.3)	45.6 (0.5)	0.94	45.2 (0.3)	45.2 (0.3)	43.7 (0.4)	0.01
Monosaccharides, E%	8.0 (0.1)	7.8 (0.1)	7.6 (0.2)	0.09	7.4 (0.1)	7.4 (0.1)	7.5 (0.2)	0.56
Disaccharides, E%	12.6 (0.2)	12.7 (0.2)	13.1 (0.3)	0.18	13.0 (0.2)	12.9 (0.2)	12.4 (0.3)	0.06
Sucrose, E%	8.5 (0.1)	8.6 (0.1)	8.8 (0.3)	0.40	8.6 (0.1)	8.5 (0.1)	8.5 (0.3)	0.51
Fiber, g/1,000 kcal	9.4 (0.1)	9.5 (0.1)	9.2 (0.2)	0.98	8.9 (0.1)	9.0 (0.1)	8.8 (0.2)	0.91
Fat, E%	38.9 (0.3)	39.1 (0.3)	39.1 (0.5)	0.51	39.3 (0.3)	39.1 (0.3)	40.8 (0.4)	0.03
Protein, E%	15.3 (0.1)	15.4 (0.1)	15.3 (0.2)	0.76	15.5 (0.1)	15.7 (0.1)	15.5 (0.2)	0.50

*The numbers represent the means (SE). A general linear model adjusting for age, sex, total energy intake, and season was used to evaluate the differences among the genotypes*.

**Table 3:** Association between the *TAS1R2* genetic variant (rs7534618) and food intake (g/day) after excluding the potential misreporters and individuals with unstable food habits.

	BMI <25				BMI ≥25			
	TT (*n* = 445)	GT (*n* = 459)	GG (*n* = 145)	*p*-Trend	TT (*n* = 470)	GT (*n* = 525)	GG (*n* = 160)	*p*-Trend
Vegetables	189 (5)	190 (4)	191 (8)	0.79	182 (5)	184 (4)	183 (8)	0.80
Fruit and berries	209 (6)	214 (6)	216 (10)	0.46	208 (6)	209 (6)	214 (10)	0.62
Potatoes	117 (3)	123 (3)	122 (5)	0.23	136 (4)	132 (3)	120 (6)	0.04
Grain/cereals (<15% sugar)	12 (1)	11 (1)	11 (1)	0.38	9 (1)	11 (1)	8 (1)	0.54
Grain/cereals (>15% sugar)	2.3 (0.4)	2.0 (0.4)	2.7 (0.6)	0.75	2.7 (0.4)	2.3 (0.4)	1.9 (0.7)	0.32
Rice and pasta	13 (1)	12 (1)	13 (1)	0.93	12 (1)	12 (1)	13 (1)	0.51
Low-fiber bread	100 (3)	92 (3)	94 (5)	0.11	102 (3)	99 (3)	90 (5)	0.07
High-fiber bread	48 (6)	58 (5)	57 (9)	0.27	40 (6)	43 (6)	71 (11)	0.03
Pastries	42 (1)	43 (1)	45 (2)	0.30	42 (2)	45 (1)	41 (3)	0.99
Sweets	6.0 (0.5)	4.8 (0.5)	4.9 (0.9)	0.13	5.1 (0.5)	5.5 (0.5)	5.0 (0.9)	0.85
Chocolate	7.8 (0.5)	8.3 (0.5)	7.9 (0.8)	0.71	8.9 (0.6)	8.5 (0.6)	11.1 (1.0)	0.18
Snacks/nuts	3.1 (0.3)	2.7 (0.3)	3.5 (0.5)	0.86	3.1 (0.3)	3.5 (0.3)	4.6 (0.6)	0.05
Jam/sugar	31 (1)	30 (1)	30 (2)	0.59	30 (1)	28 (1)	27 (2)	0.34
Fruit juice	62 (4)	59 (4)	58 (8)	0.61	55 (5)	55 (4)	69 (8)	0.20
Sugar-sweetened beverages	60 (5)	65 (5)	79 (9)	0.10	88 (7)	84 (7)	84 (12)	0.68
Low-calorie sweetened beverages	4.9 (1.4)	7.2 (1.4)	6.9 (2.5)	0.30	8.4 (2.3)	8.5 (2.2)	12.4 (3.9)	0.45
Egg	22 (1)	22 (1)	23 (1)	0.70	24 (1)	24 (1)	25 (2)	0.63
Non-processed meat	64 (2)	65 (2)	64 (3)	0.85	71 (2)	74 (2)	73 (3)	0.41
Processed meat	37 (1)	40 (1)	42 (2)	0.06	45 (1)	43 (1)	45 (2)	0.58
Poultry	16 (1)	17 (1)	16 (2)	0.96	17 (1)	17 (1)	20 (2)	0.45
Fish and shellfish	47 (2)	47 (1)	44 (3)	0.50	47 (2)	48 (2)	48 (3)	0.60
Fermented milk	107 (5)	91 (5)	94 (9)	0.06	90 (5)	92 (5)	93 (8)	0.69
Non-fermented milk	238 (10)	261 (9)	277 (17)	0.02	302 (11)	300 (10)	248 (18)	0.03
Cream	17 (1)	18 (1)	16 (1)	0.81	17 (1)	16 (1)	13 (1)	0.03
Cheese	51 (2)	48 (2)	46 (3)	0.05	44 (1)	46 (1)	49 (2)	0.04
Ice cream	13 (1)	12 (1)	12 (1)	0.63	15 (1)	15 (1)	13 (2)	0.34
Margarine	30 (1)	32 (1)	33 (2)	0.06	35 (1)	34 (1)	29 (2)	0.03
Butter-based fat	12 (1)	11 (1)	10 (2)	0.22	11 (1)	9 (1)	15 (1)	0.09
Oil/mayo/dressing	6.9 (0.4)	6.5 (0.4)	6.3 (0.7)	0.34	6.7 (0.4)	6.6 (0.4)	7.4 (0.7)	0.52

*The numbers represent the means (SE). A general linear model adjusting for age, sex, total energy intake, and season was used to evaluate the differences among the genotypes*.

## Discussion

Our results suggest that overweight and obese individuals carrying the *TAS1R2* rs7534618 T allele have a diet higher in carbohydrates. However, no significant association was observed between the sucrose intake and genotype. The higher carbohydrate intake among the major allele carriers in the group with a BMI of 25 or above is consistent with the findings reported by Dias et al. Among eight tag SNPs, these authors identified that one SNP, which is strongly linked to the SNP examined in our study (*r*^2^ = 0.87; *D*’ = 1.00), was associated with sugar sensitivity and carbohydrate intake (9). However, the magnitudes of the differences in the dietary intakes across the genotypes in our study substantially differed from those reported by Dias et al. These authors observed that carriers of the major allele (*n* = 152) had a 29% higher carbohydrate intake (*p* = 0.03) than individuals homozygous for the minor allele (*n* = 17). We only observed a 3% difference across the genotype groups in the present study. Furthermore, Dias et al. found a 38% higher sucrose intake (*p* = 0.009) in carriers of the major allele (9), which was not observed in the present study. These authors also reported a significant association between sugar sensitivity and genotype in a smaller study sample (*n* = 91). In their study, a younger population (20–29 years) was investigated in Toronto, ON, Canada. The authors used a 196-item semiquantitative food frequency questionnaire covering the previous month to assess the dietary intake. The estimated diet composition of carbohydrates and sugar was somewhat higher in the study by Dias et al. compared with the MDC cohort. In addition, according to national food intake data, somewhat higher diet composition of sugar and carbohydrates in Canada compared with Sweden has been observed (18, 19). However, the degree of overweight and obesity is comparable between the two countries (20). Both genetic variations in the sensitivity to sweetness and environmental factors affect intake and food preferences (4, 7), and genetics might have a greater impact on taste sensitivity in younger individuals. For example, obesity genetics have been shown to more strongly affect dietary intake in younger individuals (21, 22). Smokers were excluded from both studies because these individuals may have an impaired taste sensitivity (11).

In the study by Dias et al., the relationship between sugar sensitivity, sugar intake, and genotype was the opposite in participants with a BMI below 25; the carriers of the major allele (*n* = 494) had a lower carbohydrate (*p* = 0.03) and total sugar (*p* = 0.04) intake than the participants who were homozygous for the minor allele (*n* = 44) (9). We observed no significant associations across the genotypes in the group of individuals with a BMI below 25.

The misreporting of dietary intake is always possible and can influence the ability to observe true associations. Overweight individuals tend to under-report their energy intake relative to individuals with a normal weight (23). Although the dietary assessment method used in the MDC study was developed to capture the dietary habits of this population and has a high relative validity, the potential effect of this SNP on dietary habits might be too small to observe any significant differences in this cohort. The participants who reported that they changed their diets in the past were excluded because the taste sensory system may have been affected and because these participants are more aware of their diet. After excluding the smokers and individuals who were suspected of having less reliable diet reports, stronger associations were observed between genotype and carbohydrate intake.

The loci have not been identified in genome-wide association studies for macronutrients (24, 25), which supports the need for stratifying by BMI. Studies have shown that leptin, which is produced by adipocytes and regulates food intake and body weight, can affect sugar sensitivity by interacting with sweet taste receptors (26). Because obese individuals tend to have higher circulating leptin levels and are often resistant to leptin (27), individuals with a BMI of 25 or above may differ in their sensitivity to sweetness depending on their leptin levels. Therefore, leptin levels, in addition to adiposity, may be important to consider in future studies.

An impaired diet quality can lead to weight gain and impact blood lipids and the development of type 2 diabetes. Studies have shown that increased sugar intake leads to increased weight, blood pressure, elevated blood concentrations of total cholesterol, triglycerides, and LDL-C (28, 29). Because genetic variation can affect metabolism throughout an individual’s life span, genetic variations in *TAS1R2* might affect the lifetime exposure to lower sugar sensitivity and, thereby, a higher risk of developing cardiometabolic diseases. Therefore, although we were unable to observe statistically significant differences in sugar intake across the genotype carriers, a difference might exist in the prevalence of risk factors among the *TAS1R2* genotype carriers.

Several studies have examined the associations between dietary intake and other SNPs in *TAS1R2*. In a study conducted in West Mexico, the authors found an association between *TAS1R2* rs35874116 and carbohydrate intake and the risk of hypertriglyceridemia (30). Eny et al. showed an association between another genetic variation (r17879973) in *TAS1R2* and the consumption of sugar among 1,037 adolescents who completed a 196-item food frequency questionnaire. However, the association between genotype and sugar consumption was only significant in adolescents with a BMI over 25 (5). Fushan et al. (4) identified two SNPs, i.e., rs307355 and rs3574481, in the *TAS1R3* promoter region that were associated with the taste sensitivity to sucrose and explained 16% of the population’s variability in the perception of sugar. Other components in addition to sweet taste receptors can influence the sensitivity to sugar. According to a study conducted by Fushan et al., a *GNAT3* polymorphism explains 13% of sucrose perception (3). *GNAT3* encodes gustducin, which is a component of the intracellular signaling pathway for sweet, bitter, and umami tastes (3). Simultaneously studying several polymorphisms in genes encoding proteins involved in sweet taste perception and sensitivity, including *GNAT3, TAS1R1, TAS1R2*, and *TAS1R3*, would be useful.

The results of this study suggest that the dietary composition of carbohydrates and fat differed depending on the *TAS1R2* genotype. In overweight and obese individuals (BMI ≥ 25), the rs7534618 (a proxy for rs12033832) TT/GT genotypes were associated with a higher carbohydrate intake and lower fat intake than the GG genotype. Although the higher intake of carbohydrates among the major allele carriers was consistent with the results of a previous study, the magnitudes of the associations were substantially smaller. Because we observed no associations with sucrose, this allele is unlikely to be used as a marker of sugar intake in this population.

## Ethics Statement

The participants signed a written consent form, and ethical permission is available for the Malmo Diet and Cancer study (LU 90-51).

## Author Contributions

ES initiated the project and came up with the hypothesis. CH and ES analyzed data and performed the statistical analyses. CH wrote the first draft of the paper. All authors read and contributed to the interpretation and revision of the manuscript and approved the final content of the manuscript.

## Conflict of Interest Statement

The authors declare that the research was conducted in the absence of any commercial or financial relationships that could be construed as a potential conflict of interest. The reviewer CS declared a past co-authorship with one of the authors ES to the handling editor.
