# Supplementary material for: Variation in the Sweet Taste Receptor Gene and Dietary Intake in a Swedish Middle-Aged Population
Source: Front Endocrinol (Lausanne). 2017 Dec 13;8:348. doi: 10.3389/fendo.2017.00348 (PMC5733356; doi:10.3389/fendo.2017.00348)
Supplement: Supplementary file 1 [file table_1.docx]

**Supplemental Table 1. Associations between the *TAS1R2* genetic variant** (**rs7534618) and dietary intake in the entire study sample (n=3,602)**

|  | BMI<25 | | | | BMI≥25 | | | |
| --- | --- | --- | --- | --- | --- | --- | --- | --- |
|  | TT  (n=660) | GT  (n=742) | GG  (n=228) | p-  trend | TT  (n=801) | GT  (n=897) | GG  (n=274) | p-  trend |
| Energy, kcal/day | 2268 (22) | 2319 (21) | 2294 (36) | 0.29 | 2313 (21) | 2271 (20) | 2305 (35) | 0.62 |
| Carbohydrates, E% | 46.2 (0.2) | 46.2 (0.2) | 46.5 (0.4) | 0.52 | 45.9 (0.2) | 45.7 (0.2) | 44.8 (0.3) | 0.01 |
| Monosaccharides, E% | 8.2 (0.1) | 8.1 (0.1) | 8.3 (0.2) | 0.99 | 7.7 (0.1) | 7.7 (0.1) | 7.7 (0.2) | 0.99 |
| Disaccharides, E% | 12.6 (0.1) | 12.6 (0.1) | 13.0 | 0.32 | 13.0 (0.1) | 12.8 (0.1) | 12.7 (0.2) | 0.07 |
| Sucrose, E% | 8.5 (0.1) | 8.5 (0.1) | 8.8 (0.2) | 0.30 | 8.5 (0.1) | 8.3 (0.1) | 8.5 (0.2) | 0.39 |
| Fiber, g/1,000 kcal | 9.8 (0.1) | 10.1 (0.1) | 9.9 (0.2) | 0.24 | 9.4 (0.1) | 9.5 (0.1) | 9.4 (0.2) | 0.74 |
| Fat, E% | 38.2 (0.2) | 38.2 (0.2) | 38.2 (0.4) | 0.92 | 38.2 (0.2) | 38.2 (0.2) | 39.1 (0.4) | 0.09 |
| Protein, E% | 15.6 (0.1) | 15.5 (0.1) | 15.3 (0.2) | 0.31 | 15.9 (0.1) | 16.1 (0.1) | 16.1 (0.1) | 0.08 |
| Dietary changers (%) | 21.2 | 27.2 | 26.8 | 0.03 | 25.4 | 26.7 | 26.6 | 0.82 |
| Adequate energy reporters (%) | 84.7 | 84.2 | 83.3 | 0.54 | 77.5 | 78.3 | 78.5 | 0.52 |

Numbers represent the means (SE, standard error). A general linear model adjusting for age, sex, total energy intake, and season was used to evaluate the differences among the genotypes.
